# Global prevalence estimates of diffuse idiopathic skeletal hyperostosis: a systematic review and meta-analysis

**DOI:** 10.3389/fendo.2025.1517168

**Published:** 2025-05-15

**Authors:** Rui Weng, Haiwei Guo, Le Ma, Tianye Lin, Wenjun Han, Xianxing Zhong, Caijun Liu, Yikai Li, Genfu Zhu, Xuecheng Huang

**Affiliations:** ^1^ School of Traditional Chinese Medicine, Southern Medical University, Guangzhou, Guangdong, China; ^2^ The Third Affiliated Hospital of Guangzhou University of Chinese Medicine, Guangzhou, Guangdong, China; ^3^ Guangdong Research Institute for Orthopedics and Traumatology of Chinese Medicine, Guangzhou, Guangdong, China; ^4^ Shenzhen Hospital (Futian) of Guangzhou University of Chinese Medicine, Shenzhen, Guangdong, China

**Keywords:** global prevalence, diffuse idiopathic skeletal hyperostosis, systematic review, meta-analysis, DISH

## Abstract

**Background:**

Diffuse idiopathic skeletal hyperostosis (DISH) is a whole-body disease characterized by ossification or calcification of joints and ligaments, which is present on all continents and in all ethnic groups. However, there is a lack of comprehensive information on the global prevalence and incidence of DISH.

**Objective:**

To conduct a systematic review and meta-analysis to investigate the global prevalence of DISH.

**Methods:**

Three electronic medical databases (Cochrane Database of Systematic Reviews, MEDLINE, and Embase) were used to conduct a systematic review of population-based and clinical-based studies reporting the prevalence of DISH from the time of commencement to February 2023. “Prevalence or epidemiology” and “diffuse idiopathic skeletal hyperostosis or DISH” were the search terms used. There were no language restrictions. Extract data based on features such as continent, gender, age, and race. Quality was assessed using the Critical Appraisal Tool for Prevalence Data Reporting Studies from the Joanna Briggs Institute, which synthesizes the available evidence using a random effects model.

**Results:**

Among the 33 studies, the overall estimated prevalence of DISH in the general population (n=36925) was 11.92% (95% CI, 8.68%-15.59%), and the overall prevalence of DISH in clinical patients (n=22969) was about 14.30% (95%CI, 10.10%-19.09%). In 17 population-based studies, the prevalence of DISH was 10.07% (95% CI, 6.76%-13.95%) in Asia, 11.16% (95% CI, 6.19%-17.36%) in Europe, 13.46% in North America (95%CI, 12.20%-14.77%) and 30.07% (95%CI, 25.90%-34.49%) in Oceania. The overall prevalence of DISH by sex was 6.49% (95%CI, 3.65%-10.07%) in women and 17.87% (95%CI, 13.27%-22.98%) in men. The prevalence rate of Asians was 10.07% (95%CI, 6.76%-13.95%), that of white people was 11.90% (95%CI, 7.62%-16.98%), and that of black people was 8.77% (95%CI, 6.39%-11.67%). In 16 clinic-based studies, the prevalence of DISH was 16.32% (95%CI, 10.10%-23.67%) in Asia, 13.20% (95%CI, 9.89%-16.92%) in Europe, and 13.13%(95%CI, 3.79%-26.93%) in North America and 3.93% in Africa. According to gender classification, the overall prevalence of DISH was 10.16% (95%CI, 6.59%-14.38%) in women and 18.73% (95%CI, 12.84%-25.44%) in men. The prevalence rate of Asians was 16.45% (95%CI, 7.45%-28.05%), that of white people was 14.95% (95%CI, 10.28%-20.31%), and that of black people was 5.71% (95%CI, 2.57%-9.98%).

**Conclusions:**

This study identifies the global prevalence of DISH in terms of population distribution, space, and time. The overall prevalence of DISH was approximately 11.92% (95%CI, 8.68%-15.59%) in the general population and 14.30% (95% CI, 10.10%-19.09%) in clinical patients. The prevalence of DISH was higher in males, and those aged 50 and over.

## Introduction

Diffuse idiopathic skeletal hyperostosis (DISH) is an easily overlooked systemic disease characterized by ossification or calcification of spinal ligaments and entheses (1). Predominantly affecting the lower thoracic and thoracolumbar regions of the spine, DISH can also extend to the peripheral skeleton, causing hyperplastic joint changes and a decrease in mobility (2).The cause of DISH remains obscure and is suspected to involve a combination of genetic factors, metabolic processes, and inflammatory mechanisms (1, 3). The hallmark of this disease is the development of new bone, partly in entheses (3).

**Figure 1:**
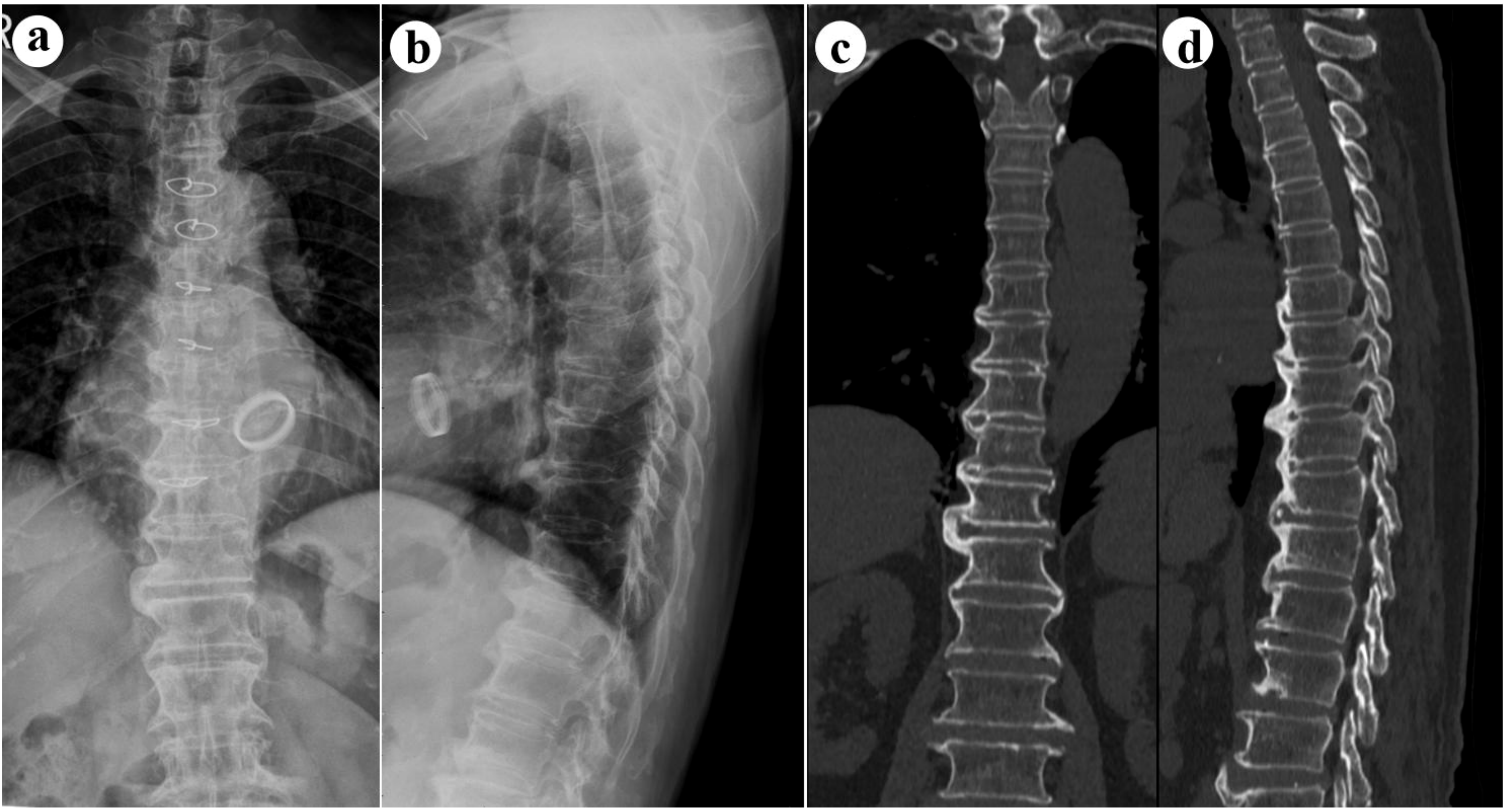
The characteristic radiological features of DISH. **(a)** An anterior-posterior view radiograph displays right-sided continuous ossification across the thoracic spine; **(b)** A lateral view radiograph reveals the presence of bony bridges anterior to the thoracic spine; **(c)** A coronal CT image depicts right-sided continuous ossification of the thoracic spine; **(d)** A sagittal CT image showcases bony bridges located in front of the thoracic spine. DISH indicates Diffuse Idiopathic Skeletal Hyperostosis; CT indicates Computed Tomography.

Forestier and Rotes-Querol initially characterized DISH in 1950, referring to it as “ankylosing hyperostosis” (4). Subsequently, Resnick and colleagues established diagnostic criteria for DISH, which include the presence of calcification along the anterolateral aspects of at least four consecutive vertebral bodies, maintenance of the intervertebral disc space, and absence of sacroiliac and apophyseal joint fusion or erosion (5). A definitive diagnosis of DISH is confirmed through the identification of these specific spinal morphological changes on imaging studies (**Figure 1**). Since DISH is predominantly asymptomatic and many individuals are not cognizant of its early presence, it has not been widely studied by medical professionals. However, in recent years, more and more evidence has shown that DISH is an indicator for a variety of pathological states, such as diabetes, hyperinsulinemia, dyslipidemia, and hyperuricemia have been reported (6–9). Furthermore, the bone formation associated with DISH may result in alterations to the musculoskeletal system’s biomechanics and the development of obstructive cervical masses (10, 11). Additionally, several studies have identified a correlation between DISH and incidents of vertebral fractures and cerebrovascular events (12, 13).

There is a scarcity of data regarding the worldwide incidence of DISH. Consequently, we conducted a systematic review and meta-analysis of existing literature with the aims: (1) to determine the global prevalence of DISH among both the general population and clinical patients; (2) to investigate the prevalence in relation to potential correlates, including publication year, geographical region, gender, age distribution, and race.

## Methods

### Literature search

Within this systematic review and meta-analysis, a comprehensive literature search was conducted from the beginning of record-keeping up to February 2023, utilizing three electronic medical databases: the Cochrane Database of Systematic Reviews, MEDLINE, and Embase. The search adhered to the guidelines set forth by the Preferred Reporting Items for Systematic Reviews and Meta-Analyses (PRISMA). The search queries encompassed terms such as “diffuse idiopathic skeletal hyperostosis or DISH” along with “prevalence or epidemiology” (For a complete outline of the search strategy, refer to **Appendix 1** in the **Supplementary Material**). The search was not limited by language, and the final iteration of the search was completed on February 20, 2023.

### Inclusion and exclusion criteria

Pre-specified inclusion criteria for study selection were as follows:

1. Studies involving epidemiological surveys or original research endeavors;
2. Studies of an observational nature, employing designs such as cross-sectional, case-control, or cohort studies;
3. Studies that provided detailed prevalence data on DISH within either the general population or clinical patient groups.

In instances where multiple studies reported on overlapping populations, only the study with the larger sample size was considered for inclusion.

The study exclusion criteria were as follows:

1. Studies such as review articles, case reports, protocols, short correspondence, letters, posters, conference abstracts or laboratory studies were excluded;
2. The reported data were insufficient, and the contact with the author is unsuccessful;
3. Studies that had no relevance to patients with DISH were excluded.

### Data extraction

The retrieved studies underwent a screening process to assess their relevance based on titles and abstracts. Subsequently, the full texts of the titles and abstracts deemed relevant were examined thoroughly. Two of our participants conducted the information extraction from the selected studies independently and then cross-verified the collected data to guarantee its precision and thoroughness. If disagreements arose, they were resolved after discussion with a third participant. We organized data from included studies into two tables, one listing population-based studies (**Table 1**) and the other listing clinical-based studies (**Table 2**). Data collected included investigators, year of publication, country, continent, gender, sample size, prevalence estimates, study period, diagnostic criteria, etc.

**Table 1:** Characteristics of 17 population-based studies of diffuse idiopathic skeletal hyperostosis (DISH).

Source	Country	Continent	No.		Prevalence, %	Study period	Diagnosis
			n(DISH)	N(Total)			
Audunsson AB, 2021 (17)	Iceland	Europe	397 Male: 311 Female: 86	5321 Male: 2276 Female: 3045	7.46	2002-2006	Resnick’s criteria(CT)
Guiot A, 2021 (18)	France	Europe	170	782	21.74	1995-1996	Resnick’s criteria(X-ray)
Okada E, 2021 (19)	Japan	Asia	39 Male: 32 Female: 7	327 Male: 174 Female: 153	11.93	2012-2016	Resnick’s criteria(X-ray)
Pini SF, 2020 (20)	Spain	Europe	207	968	21.38	2006	Resnick’s criteria(X-ray)
Uehara M, 2020 (21)	Japan	Asia	66 Male:51 Female: 15	411 Male:202 Female: 209	16.06	2014	Resnick’s criteria(X-ray)
Liang H, 2019 (22)	China	Asia	77 Male:65 Female:12	2000 Male:1335 Female:665	3.85	2010-2013	Resnick’s criteria(CT)
Bateman M, 2018 (23)	New Zealand	Oceania	138 Male:70 Female:68	459 Male:212 Female:247	30.07	2013	Resnick’s criteria(X-ray)
Banno T, 2018 (24)	Japan	Asia	42 Male:29 Female:13	504 Male:203 Female:301	8.33	2014	Resnick’s criteria(X-ray)
Katzman WB, 2017 (25)	America	North America	214 Male:161 Female:53	1591 Male:630 Female:961	13.45	1992-1996	Resnick’s criteria(X-ray)
Fujimori T, 2016 (26)	Japan	Asia	184 Male:146 Female:38	1500 Male:888 Female:612	12.27	2006-2013	Resnick’s criteria(CT)
Kagotani R, 2015 (27)	Japan	Asia	177 Male:126 Female:51	1647 Male:573 Female:1074	10.75	2005-2007	Resnick’s criteria(X-ray)
Nardo L, 2014 (28)	America	North America	152	1128	13.48	NR	Resnick’s criteria(CT)
Haara MM, 2007 (29)	Finland	Europe	151	3568 Male:1549 Female:2019	4.23	1978-1980	Forestier’s criteria(X-ray)
Kiss C, 2002 (30)	Hungary	Europe	126 Male:84 Female:42	635 Male:307 Female:328	19.84	NR	Forestier’s criteria(X-ray)
Scutellari PN, 1992 (31)	Italy	Europe	129 Male:73 Female:56	915 Male:414 Female:501	14.10	NR	Forestier’s criteria(X-ray)
Julkunen H, 1981 (32)	Finland	Europe	453	6176 Male:3100 Female:3076	7.33	1978-1980	Julkunen’s criteria(X-ray)
Julkunen H, 1975 (33)	Finland	Europe	236 Male:138 Female:98	8993 Male:4225 Female:4768	2.62	1966-1971	Julkunen’s criteria(X-ray)

**Table 2:** Characteristics of 16 clinic-based studies of diffuse idiopathic skeletal hyperostosis (DISH).

Source	Country	Continent	No.		Prevalence, %	Study period	Diagnosis
			n(DISH)	N(Total)			
Ciaffi J, 2022 (34)	Italy	Europe	130 Male: 67 Female: 63	1012 Male: 399 Female: 613	12.85	2019-2021	Resnick’s criteria(X-ray)
Ikuma H, 2022 (35)	Japan	Asia	265 Male: 185 Female: 80	1519 Male: 831 Female: 688	17.45	2020	Resnick’s criteria(CT)
Misaki H, 2022 (36)	Japan	Asia	40 Male: 28 Female: 12	100 Male: 59 Female: 41	40.00	2010-2018	Resnick’s criteria(CT)
Yoshihara H, 2021 (37)	America	North America	255 Male: 126 Female: 129	3299 Male: 1792 Female: 1507	7.73	2019-2020	Resnick’s criteria(CT)
Kuperus JS, 2018 (38)	Netherlands	Europe	145	1367	10.61	2004-2011	Resnick’s criteria(CT)
Sirasanagandla SR, 2018 (39)	Oman	Asia	130 Male:83 Female:47	1305 Male:694 Female:611	9.96	2016	Resnick’s criteria(X-ray)
Adel H, 2018 (40)	Pakistan	Asia	128 Male:94 Female:34	416 Male:270 Female:146	30.77	2017	Resnick’s criteria(CT)
Kim BS, 2018 (41)	Korea	Asia	40 Male:27 Female:13	164 Male:85 Female:79	24.39	2001-2006	Resnick’s criteria(X-ray)
Hirasawa A, 2016 (42)	Japan	Asia	98 Male:70 Female:28	558 Male:300 Female:258	17.56	2011-2012	Resnick’s criteria(X-ray)
Mori K, 2016 (43)	Japan	Asia	261 Male:230 Female:31	3013 Male:1752 Female:1261	8.66	2010	Resnick’s criteria(CT)
Westerveld LA, 2008 (44)	Netherlands	Europe	85 Male:52 Female:33	501 Male:229 Female:272	16.97	2004-2006	Resnick’s criteria(X-ray)
Mader R, 2005 (45)	Israel	Asia	100 Male:50 Female:50	1020	9.80	NR	Resnick’s criteria(X-ray)
Kim SK, 2004 (46)	Korea	Asia	104 Male:88 Female:16	3595 Male:1616 Female:1979	2.89	2003	Resnick’s criteria(X-ray)
Weinfeld RM, 1997 (47)	America	North America	466 Male:277 Female:189	2364 Male:1107 Female:1257	19.71	NR	Resnick’s criteria(X-ray)
Cassim B, 1990 (48)	South Africa	Africa	59 Male:42 Female:17	1500 Male:1092 Female:408	3.93	NR	Resnick’s criteria(X-ray)
Bloom RA, 1984 (49)	Israel	Asia	222 Male:140 Female:82	1236 Male:624 Female:612	17.96	1981-1982	Resnick’s criteria(X-ray)

### Quality assessment

Two of our participants assessed risk of bias for included studies using the Joanna Briggs Institute Critical Appraisal Instrument for Studies Reporting Prevalence Data (**eTable 1** in the **Supplementary Material**). Discrepancies were resolved through conversation with a third participant. The Joanna Briggs Institute’s quality evaluation tool for prevalence studies includes 9 items, which evaluate the overall quality of prevalence studies in terms of sampling methods, research objects, data collection and analysis methods, etc. Each item was judged as yes, no, unclear or not applicable. The higher the total score, the better the quality of the included studies and the lower the risk of bias. Studies were classified according to the percentage of ‘yes’ responses as high quality (≥70%), moderate quality (<70% and >50%), and low quality (≤50%).

### Data analysis

Data analysis was conducted utilizing the metaprops module within the R Statistical Package (version 3.5.3). The logit method was employed to convert the reported prevalence figures from each study, which was then followed by an inverse-variance weighted random-effects meta-analysis, adopting the approach outlined by Holden et al. (14). This process allowed us to derive the prevalence of DISH, along with 95% confidence intervals (CIs), across the entire population and various subgroups. Heterogeneity among the studies was evaluated using the I^2^ statistic and the Cochrane Q statistic for heterogeneity P value, which quantifies the proportion of variability across studies attributable to heterogeneity rather than chance (15). These results were visually represented in a forest plot. The Egger test was used to assess publication bias, with the findings depicted in a funnel plot. The true extent of heterogeneity, in terms of variance or standard deviation, was indicated by τ^2^ (16). We also explored two distinct measures of residual heterogeneity, I^2^ and τ^2^, using the metafor package in R for random-effects meta-regression. The primary outcome measured was the prevalence of DISH in the general population and across different subgroups. Further subgroup analyses were conducted based on factors such as continent, gender, age, and ethnicity.

## Results

### Literature search and included studies

**Figure 2:**
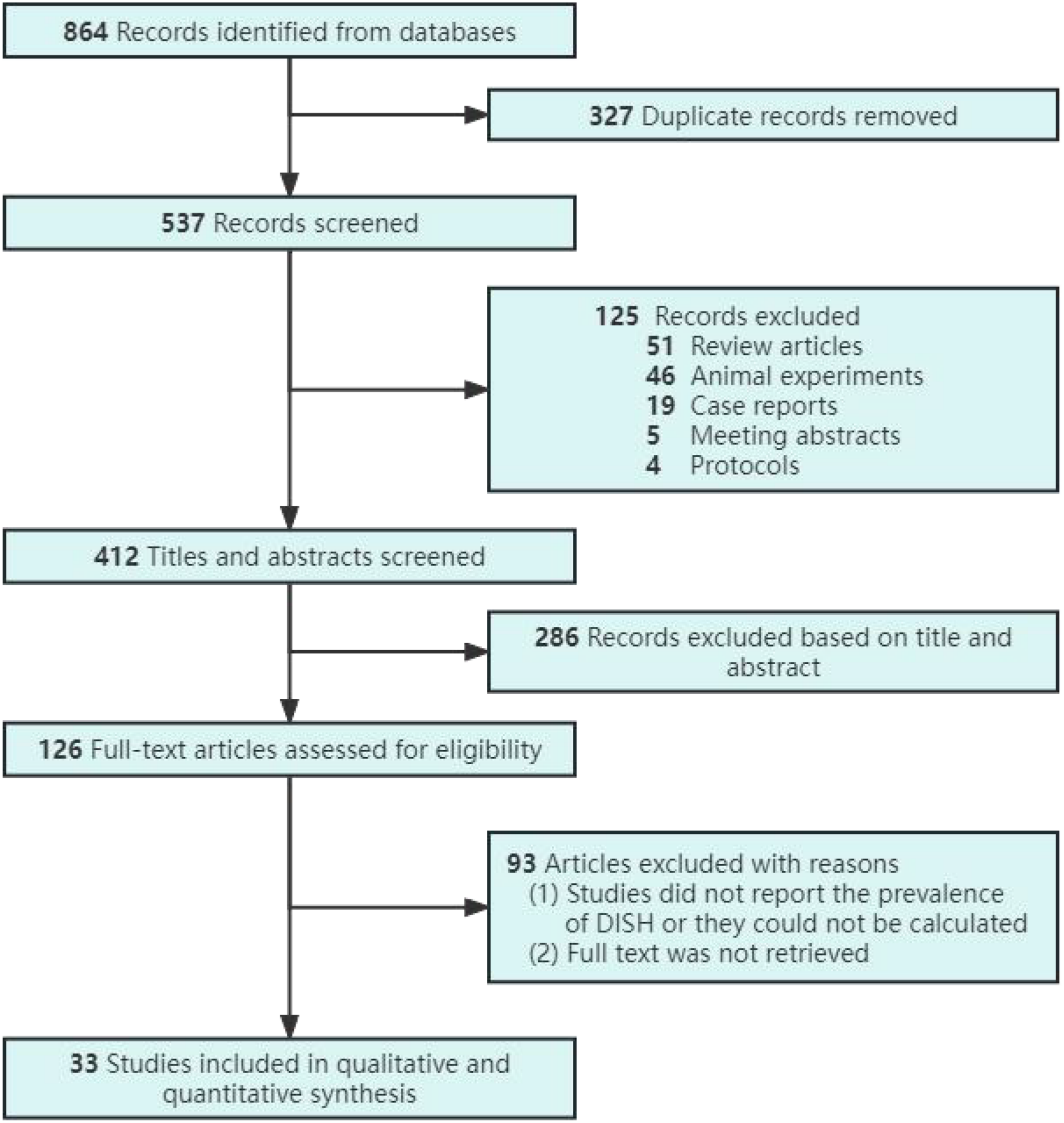
PRISMA flow diagram. DISH indicates Diffuse Idiopathic Skeletal Hyperostosis; PRISMA, Preferred Reporting Items for Systematic Reviews and Meta-analyses.

Using the search terms “diffuse idiopathic skeletal hyperostosis or DISH” and “prevalence or epidemiology”, 864 records were obtained from 3 databases. The PRISMA flowchart shows the retrieval process (**Figure 2**). A total of 33 studies were included in this search (17–49), of which 17 were population-based studies (17–33) and 16 were clinical-based studies (34–49). The 17 population-based studies represented 10 different countries (**Table 1**), 8 of which were conducted in Europe, 6 in Asia, 2 in North America, and 1 in Oceania. The 16 clinical-based studies represented 9 different countries (**Table 2**), of which 3 were conducted in Europe, 10 in Asia, 2 in North America, and 1 in Africa.

### Overall prevalence of DISH

**Figure 3:**
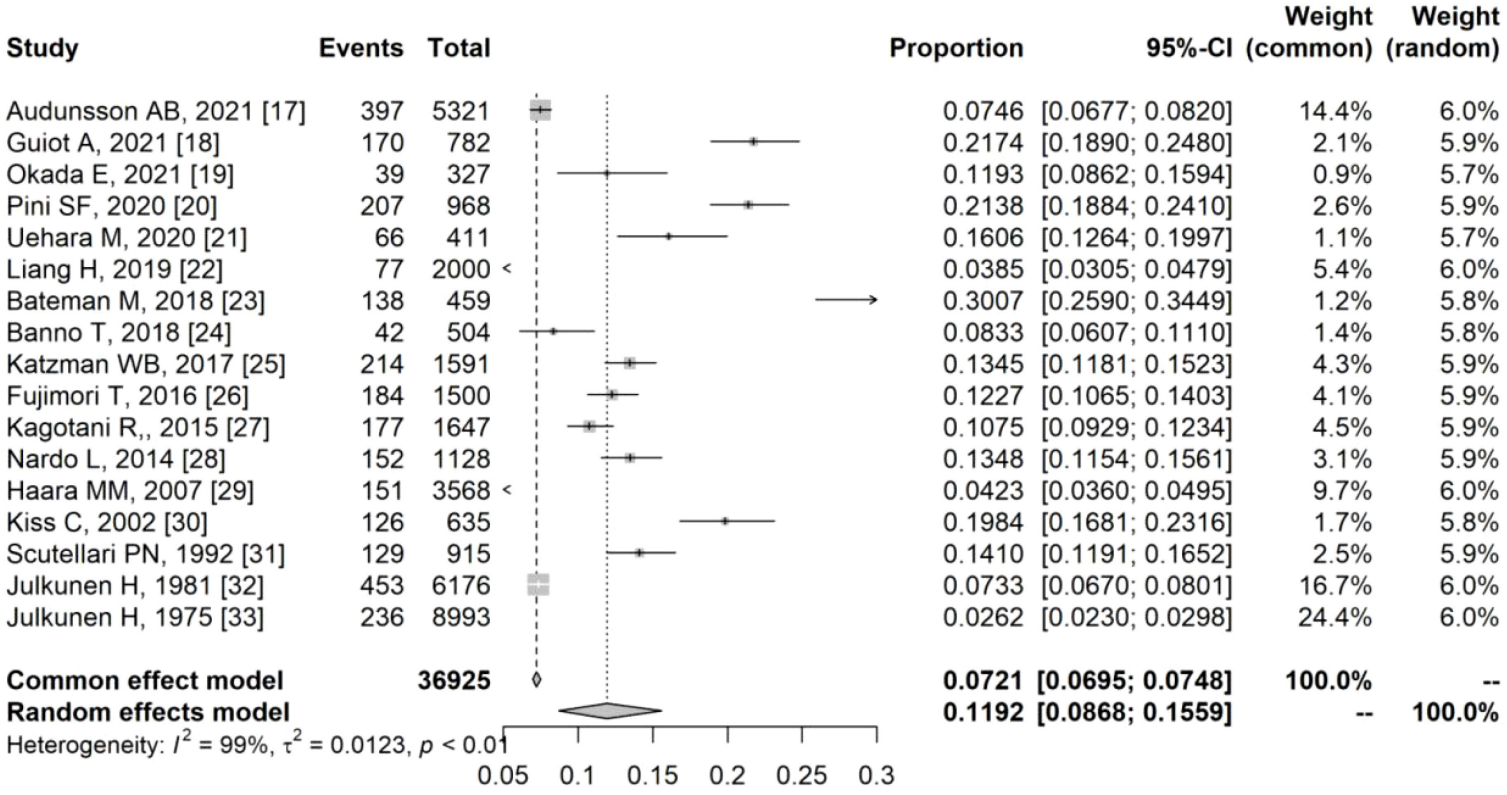
Forest plot of the overall prevalence of diffuse idiopathic skeletal hyperostosis in population-based studies.

**Figure 4:**
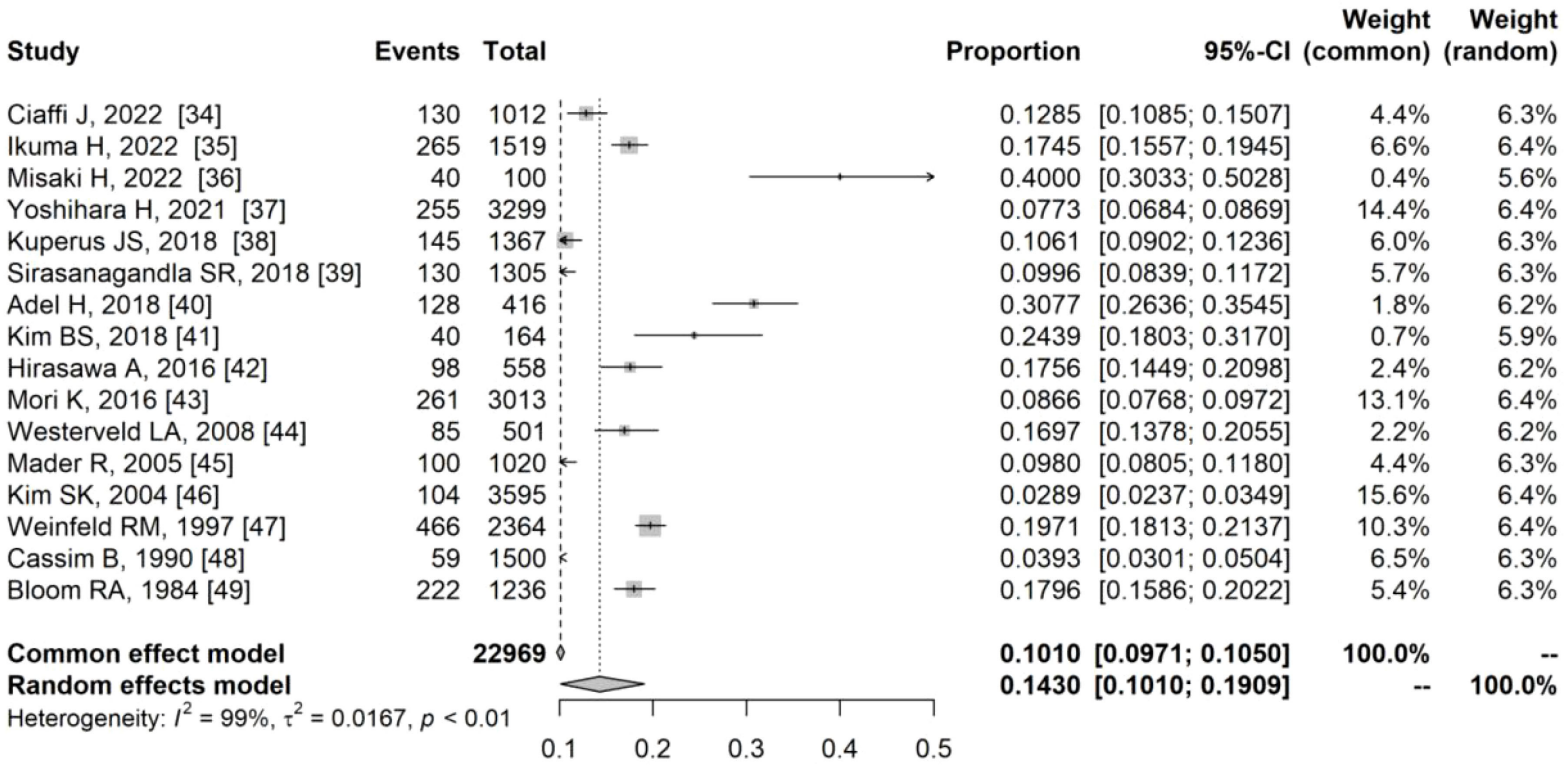
Forest plot of the overall prevalence of diffuse idiopathic skeletal hyperostosis in clinic-based studies.

The overall prevalence of DISH was calculated through meta-analysis and depicted in **Figures 3**, **4**. Prevalence estimates based on population studies (n=36925) ranged from 2.62% to 30.07% (**Figure 3**), and the overall estimated prevalence of DISH pooled by random effects was 11.92% (95%CI, 8.68%-15.59%) (I^2^ = 99%; P < 0.01). Prevalence estimates based on clinical studies (n = 22969) ranged from 2.90% to 40.00% (**Figure 4**), and the overall estimated prevalence of DISH pooled by random effects was 14.30% (95% CI, 10.10%-19.09%) (I^2^ = 99%; P < 0.01).

### Prevalence of DISH by publication year

To explore whether there was a correlation between the year and the prevalence of DISH, we performed an association analysis by year of publication (**eFigures 1**, **2** in the **Supplementary Material**). As can be seen from the figure, the prevalence of DISH fluctuates with the year of publication. The prevalence of DISH varied from 2.60% in 1975 to 30.07% in 2018 in population-based studies (17–33), while in clinical-based studies (34–49) from 2.90% in 2004 to 40.00% in 2022. However, as depicted by the scatterplot, no significant correlation was found between the year of publication and the high or low prevalence rates of DISH.

### Prevalence of DISH by continent

Among 17 population-based studies (17–33), 8 were conducted in Europe (n=27358), 6 in Asia (n=6389), 2 in North America (n=2719), and 1 in Oceania (n=459). The prevalence of DISH was 11.16% (95% CI, 6.19%-17.36%) in Europe (with very high heterogeneity [I^2^ = 99%; P < 0.01]), 10.07% (95%CI, 6.76%-13.95%) in Asia (with very high heterogeneity [I^2^ = 96%; P < 0.01]), 13.46% (95%CI, 12.20%-14.77%) in North America (I^2^ = 0%;P =0.98),and 30.07% (95%CI, 25.90%-34.49%) in Oceania (**eFigure 3** in the **Supplementary Material**).

Of the 16 clinical-based studies (34–49) most were from Asia (n=12926), only 3 were conducted in Europe (n=2880), 2 in North America (n=5663), and 1 in Africa (n=1500). The prevalence of DISH was 16.32% (95%CI, 10.10%-23.67%) in Asia (with very high heterogeneity [I^2^ = 99%; P < 0.01]), 13.20% (95%CI, 9.89%-16.92%) in Europe (with very high heterogeneity [I^2^ = 85%; P < 0.01]), 13.13%(95%CI, 3.79%-26.93%) in North America (with very high heterogeneity [I^2^ = 99%; P < 0.01]), 3.93% (95% CI,3.01%-5.04%) in Africa (**eFigure 4** in the **Supplementary Material**).

### Prevalence of DISH by sex

Gender differences in the prevalence of DISH were observed separately in 17 population-based studies and 16 clinical-based studies. Of 17 population-based studies, 12 studies reported the numbers of men and women with and without DISH, and 2 studies included men only. A total of 12864 women (49.38%) and 13189 men (50.62%) were included in these 14 studies. The overall prevalence of DISH was 6.49% (95%CI, 3.65%-10.07%) in women (with very high heterogeneity [I^2^ = 96%; P < 0.01]) and 17.87% (95%CI, 13.27% -22.98%) in men (with very high heterogeneity [I^2^ = 99%; P < 0.01]) (**eFigure 5** in the **Supplementary Material**). Of the 16 clinical-based studies, 14 studies reported the gender prevalence of DISH, with 9732 females (47.28%) and 10850 males (52.72%). The pooled overall prevalence of DISH was 10.16% (95%CI, 6.59%-14.38%) in women (with very high heterogeneity [I^2^ = 98%; P < 0.01]) and 18.73% (95%CI, 12.84% -25.44%) in men (with very high heterogeneity [I^2^ = 98%; P < 0.01])(**eFigure 6** in the **Supplementary Material**). Men appear to have a higher prevalence of DISH than women.

### Prevalence of DISH by race

Racial differences in the prevalence of DISH have been observed in both population-based and clinical-based studies. Of the 17 population-based studies that clarified the original region of the participants, 1 multiethnic study did not report in detail the number of black and white people with or without DISH. Among the 16 studies included studies, the prevalence of DISH was 11.90%(95%CI, 7.62% to 16.98%) in white race (with very high heterogeneity [I^2^ = 99%; P < 0.01]), 10.07% (95%CI, 6.76%-13.95%) in Asian race (with very high heterogeneity [I^2^ = 96%; P < 0.01]), and 8.77% (95%CI, 6.39%-11.67%) in black race (**eFigure 7** in the **Supplementary Material**). Of the 16 clinical-based studies, 1 study involving multiple races did not report in detail the number of Asian, black, and white race with or without DISH. In the remaining 15 studies, the prevalence of DISH was 5.71% (95%CI, 2.57%-9.98%) in black race (with very high heterogeneity [I^2^ = 96%;P < 0.01]), 16.45% (95% CI, 7.45% to 28.05%) in Asian race (with very high heterogeneity [I^2^ = 99%; P < 0.01]), and 14.95%(95%CI, 10.28%-20.31%) in white race (with very high heterogeneity [I^2^ = 96%; P < 0.01]) (**eFigure 8** in the **Supplementary Material**). The prevalence of DISH in black race appeared to be lower than in white and Asian race. However, the number of studies in black race was limited, and more research was needed to enhance reliability.

### Prevalence of DISH by age

In the 33 studies, most of the subjects were over 30 years old. There was 1 population-based study and 7 clinical-based studies with detailed age groups. The results of 7 clinical-based studies showed that the prevalence of DISH was 2.67% (95%CI, 0.87% to 5.27%) for patients under 50 years old (with very high heterogeneity [I^2^ = 84%; P < 0.01]), 6.71% (95% CI, 3.01% to 11.61%) for patients aged 50–59 years (with very high heterogeneity [I^2^ = 94%; P < 0.01]), 12.46% (95% CI, 6.14% to 20.52%) for patients aged 60–69 years (with very high heterogeneity [I^2^ = 96%; P < 0.01]), 18.48% (95% CI, 10.50% - 28.04%) for patients aged 70–79 years (with very high heterogeneity [I^2^ = 96%; P < 0.01]), and 19.75% (95% CI, 9.93% to 31.82%) for patients over 80 years old (with very high heterogeneity [I^2^ = 93%; P < 0.01]).

### Publication bias

Among these 17 population-based studies, Egger’s test revealed a statistically significant publication bias (P < 0.05). Similarly, Egger’s test showed statistically significant publication bias among the 16 clinic–based studies (P < 0.05). Therefore, publication bias may be a source of heterogeneity.

## Discussion

To our knowledge, this systematic review and meta-analysis represents the inaugural effort to assess the worldwide prevalence of DISH among both the general population and clinical patients. Within this study, we examined the global prevalence of DISH across various spatial, temporal, and demographic dimensions. The findings revealed an overall estimated prevalence of DISH of 11.92% within the general population and 14.30% among clinical patients. Additionally, this research indicates that men exhibit a higher prevalence of DISH in comparison to women. It mainly affects people aged 50 and over, and it tends to increase with age. In addition, the prevalence of DISH in black race is lower than that in white and Asian race. However, the number of studies in black race was limited, and more research was needed to enhance reliability. No statistical association was observed between the prevalence of DISH and year of publication. Due to the high heterogeneity of the included studies, which may be attributed to differences in study design, population characteristics, and diagnostic criteria, our findings should be interpreted with caution.

There is high heterogeneity among studies on the prevalence of DISH for several possible reasons. First, because DISH is, for the most part, an asymptomatic disease, most patients do not seek medical help, and the diagnosis of this disease is usually established during an examination of other disorders. Therefore, it is difficult to reliably estimate the prevalence of DISH in the population, which inevitably involves chance factors. In addition, the criteria for establishing the diagnosis of DISH are not always uniformly applied. The Resnick and Niwayama criteria of 1976 called for a “bone bridge” connecting at least 4 adjacent vertebrae, or ossification of the anterior longitudinal ligament, while maintaining intervertebral height, with no spondyloarthritis or sacroiliac joint changes (5). On the basis of the former, some scholars also lowered the standard to three adjacent vertebral bodies or there may be incompletely connected bone bridges between vertebral bodies (50). In addition, some scholars have also included factors such as the influence of surrounding bones, because the manifestations of DISH are not limited to the spine, and peripheral joints and joint capsules are often affected (51). Indeed, ossification and/or calcification near peripheral joints, such as tendons, ligaments, and joint capsules, can be observed in patients with DISH (52, 53). What’s more, some diseases that manifest as excessive bone formation and bone involvement can easily confuse the diagnosis (54), such as ankylosing spondylitis, ossification of the anterior longitudinal ligament, etc. These diseases all have a tendency to ossify ligaments or joints, and it is not always easy to distinguish these diseases. In addition, the choice of imageological examination also has an impact, such as choosing X-ray or CT examination, choosing thoracic spine or whole spine (34). In previous reports, the prevalence of DISH identified only by x-ray was lower than that using CT (42). Finally, methodological heterogeneity should also be taken into account, as the prevalence data came from different study designs with different levels of methodological quality. Examples include different study populations, sampling methods and coverage of samples, sample size, imageological examinations, data collection, participant collaboration, and examiner expertise, resulting in differences between studies.

Overall, our study found a higher prevalence in clinical patients than in the general population. As previously described, DISH is in most cases an asymptomatic disease that can be difficult to detect without appropriate testing. Another reason is that DISH disease has been found to be significantly associated with metabolic diseases (such as diabetes, obesity, and hyperuricemia), fractures, and low back pain, and because the effects of these diseases are detected at the time of care, clinical patients have a relatively higher prevalence than the general population. Our study also found that, according to different populations, races, ages, sexes, and imaging studies, the prevalence of DISH varied greatly among different studies (**Tables 1**, **2**).

Subgroup analysis by sex showed a higher prevalence of DISH in men than in women, unlike many types of arthritis. One of the reasons for this discrepancy may be related to manual labor. DISH, similar to osteophyte formation, is considered to be a response of the bone to stress or repeated microtrauma, and it is mainly seen in manual laborers, especially men (55). Julkunen et al. (32) analyzed the occupation of their patients and found that heavy physical labor may increase the chance of disease, however, further research is still needed to fully understand the underlying mechanisms. Subgroup analysis by age showed that DISH diseases mainly affected people aged 50 years and over, and there was an increasing trend with age. One of the reasons may be related to the continuous development of the formation of DISH. The onset process of DISH begins in early life, but it takes decades for the disease to fully develop and meet the diagnostic criteria (3). Another possible reason is related to multiple metabolic factors, which are widely recognized as key determinants in patients with DISH. Known risk factors such as obesity, diabetes and hormones themselves are also associated with aging. Obesity is closely associated with DISH as well as other widespread musculoskeletal disorders (56, 57), and the role of adipokines in the pathogenesis of DISH has been proposed because they affect bone metabolism and promote the number and activity of osteoblasts (58, 59). Several studies analyzing the link between DISH and diabetes have suggested that a possible pathogenesis is that high levels of insulin or insulin-like growth factor stimulate new bone formation (60). In fact, insulin is a peptide that promotes bone growth (61) and is considered a key player in the pathophysiology of DISH and other spinal inflammatory and degenerative diseases (62). In addition, hormones or growth factors are also involved in the pathogenesis of DISH because they act on fibroblasts, chondrocytes, and collagen fibers of cartilage to promote bone regeneration (63).

Subgroup analysis by race showed that the Black race has a lower incidence of DISH than the White race and the Asian race. This may be because the Black race possesses some specific gene sequences or gene expression patterns, which influence physiological processes related to the pathogenesis of DISH, such as bone metabolism and cell signaling pathways. Certain genes may endow the bones of Black individuals with a stronger resistance to the pathological processes leading to DISH, reducing the tendency of bone spur formation and ligament calcification, thus decreasing the occurrence of DISH. Another reason is that Black people may face a shortage of healthcare resources, which may lead to a relatively higher rate of missed diagnosis or misdiagnosis of DISH. Some early-stage or mildly symptomatic DISH cases may not be detected and diagnosed in a timely manner. In addition, due to their relatively disadvantaged socioeconomic status and the relative scarcity of medical facilities, the conduct of clinical research is limited. Considering cultural and ideological factors as well, there is relatively less research on the Black race compared to the White and Asian races.

At present, the pathogenesis of DISH is still unclear. It is increasingly recognized that the development of DISH disease is progressive continuously (64). From the microscopic level, the most important feature in the early stage is the formation of new bone, which progressively forms a bony bridge from one vertebral body to the adjacent vertebral body, accompanied by biomechanical changes (65). According to Wolff’s law, bone is continuously remodeled under the influence of biomechanical loading (66). Over time, under long-term gravitational loading, the bone bridge may gradually integrate the upper and lower vertebral bodies. New bone is mainly formed in the epiphysis, and local fibroblasts, chondrocytes, collagen fibers, and calcified matrix can promote the formation of new bone, but it is affected by genetic, vascular, metabolic, and mechanical factors (67). In addition, diseases related to modern lifestyles, such as obesity and type 2 diabetes, have been associated with the development of DISH (56, 60), which has emerged in Western European countries such as the United Kingdom. As DISH is frequently found to be comorbid with metabolic disorders, DISH may be driven at least in part by biochemical pathways shared with metabolic disorders such as hyperlipidemia and hyperglycemia (19). In addition, it is also related to genetics. Sethi et al. (68) identified 10 DISH-related loci through genetic association analysis, including multiple genes related to bone remodeling, such as osteogenesis master regulator RUNX2, BMP signaling pathway (CHRDL2, NOG, GDFS), wnt signaling pathway (ROR2) and ILI1 etc., implying that overactive osteogenesis plays an important role in the disease development of DISH.

In clinical practice, it has been found that DISH has a direct correlation with fractures (13, 69). Continuously fused spinal segments in patients with DISH resemble long bones, and the number of fused segments determines the length of the “lever arm” over which traumatic forces can act. Longer lever arms can cause severely displaced spinal fractures after relatively minor trauma, such as a fall from standing or sitting position or a low-speed motor vehicle crash (39). On the other hand, it is well known that the reduction of intravertebral bone mineral density is inversely proportional to the extravertebral bone hyperplasia over time (70). When attempting to assess bone mineral density using dual-energy X-ray absorptiometry (DEXA), the occurrence of extravertebral hyperostosis can lead to markedly increased bone density, making it difficult to assess intravertebral osteoporosis.

Therefore, this may hamper the appropriate treatment of osteoporosis in DISH patients, making patients more prone to spinal fractures. As mentioned above, many of the problems associated with DISH observed in the spine have been noted so far, but few large epidemiological investigations of this disease have been reported. Knowledge of the epidemiology and associated clinical and radiological features of DISH may help improve awareness among rheumatologists, radiologists, and their associated clinicians, and allow proper recognition and reporting of the condition. In addition, we also expect that by comparing the incidence rates of populations with different exposure factors, we can identify potential causative agents and risk factors, providing data support for the rational allocation of healthcare resources and the formulation of health policies. Although the excessive bone formation in DISH is unlikely to be reversed, it may be prevented with increased knowledge. Therefore, it is necessary to conduct a large-scale epidemiological study of the disease to understand the clinical characteristics of DISH and its prevalence, natural process and outcome, which will help pave the way for more targeted and effective treatments in the future.

### Limitations

Although our study included global studies on the prevalence of DISH, some limitations need to be acknowledged. First, the heterogeneity of the included literature is very high, which may be partly explained by the population studied, diagnostic criteria, and imaging selection. Currently, there is a lack of up-to-date accepted criteria for the accurate diagnosis of the disease. Second, although our study is based on the results of 59894 individuals, we cannot directly access these databases, which prevents us from obtaining more epidemiological information, such as detailed age distribution, body mass index (BMI), relevant underlying disease information, etc. In addition, despite a number of subgroup and regression analyses, most of the heterogeneity is unexplained.

## Conclusions

This study determined the global prevalence of DISH in terms of spatial, temporal and population distribution. We estimated the overall prevalence of DISH to be approximately 11.92% (95%CI, 8.68%-15.59%) in the general population and 14.30% (95% CI, 10.10%-19.09%) in clinical patients. In addition, it was also found that the prevalence of DISH was higher in men compared with women. It mainly affects people aged 50 and over, and it tends to increase with age. Black race has a lower incidence of DISH than white race and Asian race. Due to the high heterogeneity of the included studies, our findings should be considered with caution.

## Data Availability

The original contributions presented in the study are included in the article/**Supplementary Material**. Further inquiries can be directed to the corresponding authors.
