# Supplementary material for: Global prevalence estimates of diffuse idiopathic skeletal hyperostosis: a systematic review and meta-analysis
Source: Front Endocrinol (Lausanne). 2025 May 15;16:1517168. doi: 10.3389/fendo.2025.1517168 (PMC12119254; doi:10.3389/fendo.2025.1517168)

**eAppendix 1**. Search Strategy

**eTable 1**. Quality assessment

**eFigure 1**. Association between prevalence of DISH based on population studies and publication year.

**eFigure 2**. Association between prevalence of DISH based on clinical studies and publication year.

**eFigure 3**.Forest plot of the prevalence of DISH by continent in population-based studies

**eFigure 4**. Forest plot of the prevalence of DISH by continent in clinic-based studies.

**eFigure 5**. Forest plot of the prevalence of DISH by sex in population-based studies.

**eFigure 6**. Forest plot of the prevalence of DISH by sex in clinic-based studies.

**eFigure 7**. Forest plot of the prevalence of DISH by race in population-based studies.

**eFigure 8**. Forest plot of the prevalence of DISH by race in clinic-based studies.

**eFigure 9**. Forest plot of the prevalence of DISH by age in clinic-based studies.

**eFigure 10**. Publication bias of population-based prevalence studies of DISH.

**eFigure 11**. Publication bias of clinic-based prevalence studies of DISH.

eAppendix 1. Search Strategy

1. Diffuse Idiopathic Skeletal Hyperostosis.mp. [mp=tx, bt, ti, ot, ab, ct, sh, hw, kw, tn, dm, mf, dv, fx, dq, nm, kf, ox, px, rx, an, ui, ds, on, sy]

2. DISH.mp. [mp=tx, bt, ti, ot, ab, ct, sh, hw, kw, tn, dm, mf, dv, fx, dq, nm, kf, ox, px, rx, an, ui, ds, on, sy]

3. Prevalence.mp. [mp=tx, bt, ti, ot, ab, ct, sh, hw, kw, tn, dm, mf, dv, fx, dq, nm, kf, ox, px, rx, an, ui, ds, on, sy]

4. Epidemiology.mp. [mp=tx, bt, ti, ot, ab, ct, sh, hw, kw, tn, dm, mf, dv, fx, dq, nm, kf, ox, px, rx, an, ui, ds, on, sy]

5. 1 or 2

6. 3 or 4

7. 5 and 6

eTable 1. Quality assessment

| **Author, year** | **Q1** | **Q2** | **Q3** | **Q4** | **Q5** | **Q6** | **Q7** | **Q8** | **Q9** | **Total(%)** | **Risk of Bias** |
| --- | --- | --- | --- | --- | --- | --- | --- | --- | --- | --- | --- |
| **Population-Based Studies** | | | | | | | | | | | |
| Audunsson AB, 2021 [17] | U | U | Y | N | U | Y | Y | Y | U | 44.44 | HIGH |
| Guiot A, 2021 [18] | N | Y | Y | N | N | Y | Y | Y | U | 55.56 | MODERATE |
| Okada E, 2021 [19] | N | Y | Y | Y | U | Y | U | Y | U | 55.56 | MODERATE |
| Pini SF, 2020 [20] | N | Y | Y | Y | U | Y | Y | Y | U | 66.67 | MODERATE |
| Uehara M, 2020 [21] | Y | Y | Y | Y | Y | Y | Y | Y | U | 88.89 | LOW |
| Liang H, 2019 [22] | N | Y | Y | Y | U | Y | Y | Y | U | 66.67 | MODERATE |
| Bateman M, 2018 [23] | N | U | Y | Y | U | Y | N | Y | U | 44.44 | HIGH |
| Banno T, 2018 [24] | N | U | Y | Y | Y | Y | N | Y | U | 55.56 | MODERATE |
| Katzman WB, 2017 [25] | Y | Y | Y | Y | Y | Y | Y | Y | Y | 100 | LOW |
| Fujimori T, 2016 [26] | N | Y | Y | Y | Y | Y | Y | Y | U | 77.78 | LOW |
| Kagotani R,, 2015 [27] | Y | Y | Y | Y | Y | Y | N | Y | U | 77.78 | LOW |
| Nardo L, 2014 [28] | N | Y | Y | Y | N | Y | Y | Y | Y | 77.78 | LOW |
| Haara MM, 2007 [29] | Y | Y | Y | Y | U | Y | N | Y | Y | 77.78 | LOW |
| Kiss C, 2002 [30] | Y | U | Y | Y | Y | Y | Y | Y | Y | 88.89 | LOW |
| Scutellari PN, 1992 [31] | Y | Y | Y | Y | U | Y | U | Y | U | 66.67 | MODERATE |
| Julkunen H, 1981 [32] | Y | Y | Y | Y | Y | Y | Y | Y | Y | 100 | LOW |
| Julkunen H, 1975 [33] | Y | Y | Y | Y | Y | Y | N | Y | Y | 88.89 | LOW |
| **Clinic-Based Studies** | | | | | | | | | | | |
| Ciaffi J, 2022 [34] | N | Y | Y | Y | Y | Y | Y | Y | U | 77.78 | LOW |
| Ikuma H, 2022 [35] | N | Y | Y | Y | U | Y | N | Y | U | 55.56 | MODERATE |
| Misaki H, 2022 [36] | N | Y | N | Y | N | Y | Y | Y | U | 55.56 | MODERATE |
| Yoshihara H, 2021 [37] | N | Y | Y | Y | Y | Y | Y | Y | U | 77.78 | LOW |
| Kuperus JS, 2018 [38] | N | Y | Y | Y | U | Y | Y | Y | Y | 77.78 | LOW |
| Sirasanagandla SR, 2018 [39] | N | Y | Y | Y | Y | Y | N | Y | U | 66.67 | MODERATE |
| Adel H, 2018 [40] | N | U | Y | Y | N | Y | Y | Y | U | 55.56 | MODERATE |
| Kim BS, 2018 [41] | N | U | N | Y | Y | Y | Y | Y | U | 55.56 | MODERATE |
| Hirasawa A, 2016 [42] | N | Y | Y | Y | Y | Y | Y | Y | U | 77.78 | LOW |
| Mori K, 2016 [43] | N | Y | Y | Y | Y | Y | Y | Y | U | 77.78 | LOW |
| Westerveld LA, 2008 [44] | N | Y | Y | Y | Y | Y | Y | Y | Y | 88.89 | LOW |
| Mader R, 2005 [45] | N | Y | Y | Y | N | Y | N | Y | N | 55.56 | MODERATE |
| Kim SK, 2004 [46] | N | Y | Y | Y | Y | Y | N | Y | Y | 77.78 | LOW |
| Weinfeld RM, 1997 [47] | N | Y | Y | Y | Y | Y | Y | Y | U | 77.78 | LOW |
| Cassim B, 1990 [48] | N | Y | Y | Y | U | Y | N | Y | U | 55.56 | MODERATE |
| Bloom RA, 1984 [49] | N | Y | Y | Y | U | Y | N | Y | U | 55.56 | MODERATE |

Legend: Y=Yes; N=No; U=Unclear; NA=Not applicable

Q1: Was the sample frame appropriate to address the target population?

Q2: Were study participants sampled in an appropriate way?

Q3: Was the sample size adequate?

Q4: Were the study subjects and the setting described in detail?

Q5: Was the data analysis conducted with sufficient coverage of the identified sample?

Q6: Were valid methods used for the identification of the condition?

Q7: Was the condition measured in a standard, reliable way for all participants?

Q8: Was there appropriate statistical analysis?

Q9:Was the response rate adequate, and if not, was the low response rate managed appropriately?

Total= ΣY/ Apllicable Items (the Not Applicable (NA) items were excluded from the sum).

Risk of bias was categorized as high when the study reaches up to 49% score “yes”, moderate when the study reached 50% to 69% score “yes”, and low when the study reached more than 70% score “yes”.

eFigure 1. Association between prevalence of DISH based on population studies and publication year.


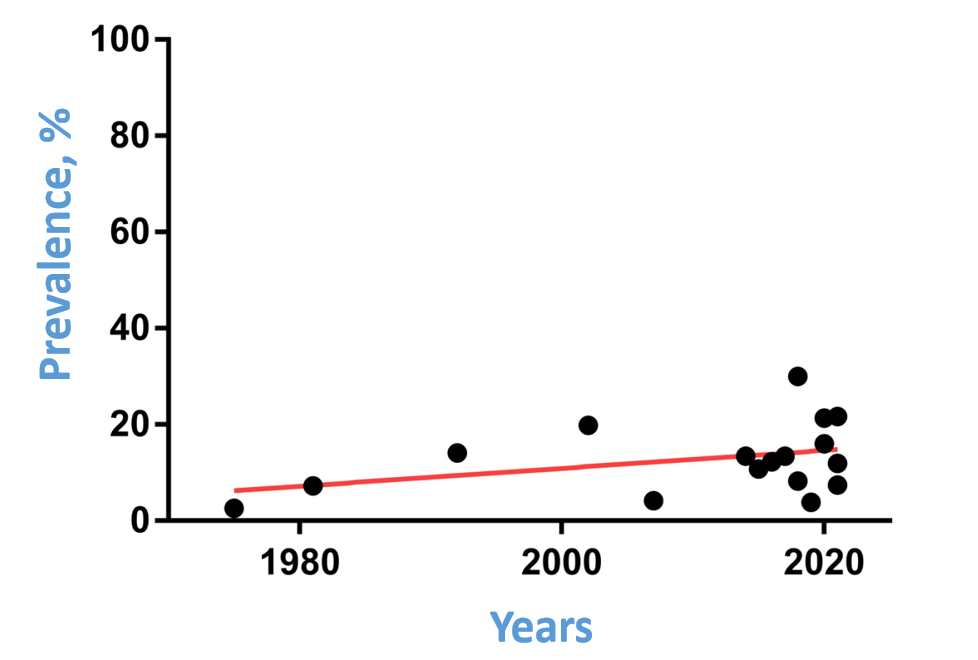


eFigure 2. Association between prevalence of DISH based on clinical studies and publication year.


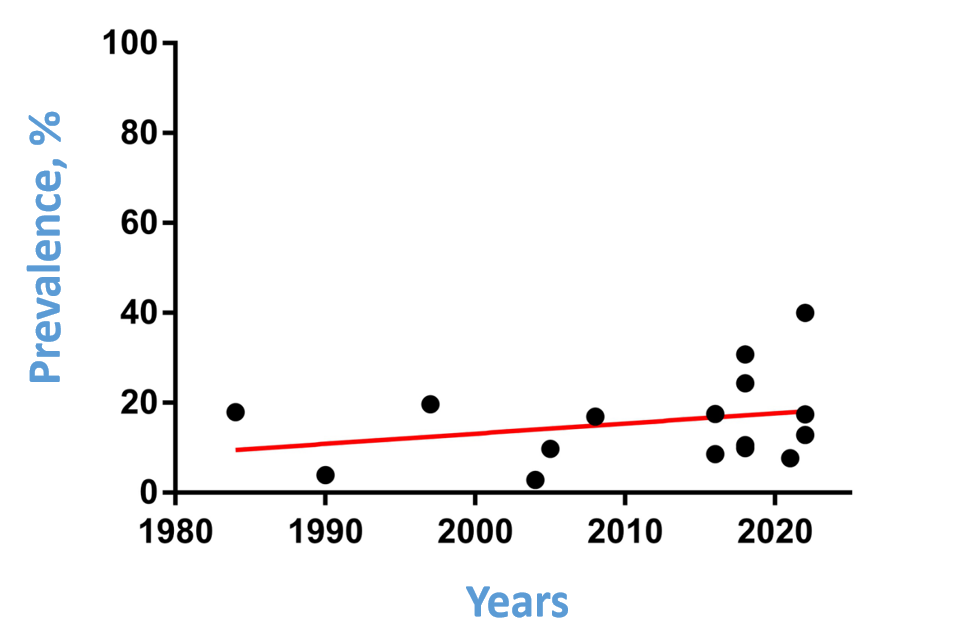


eFigure 3. Forest plot of the prevalence of DISH by continent in population-based studies


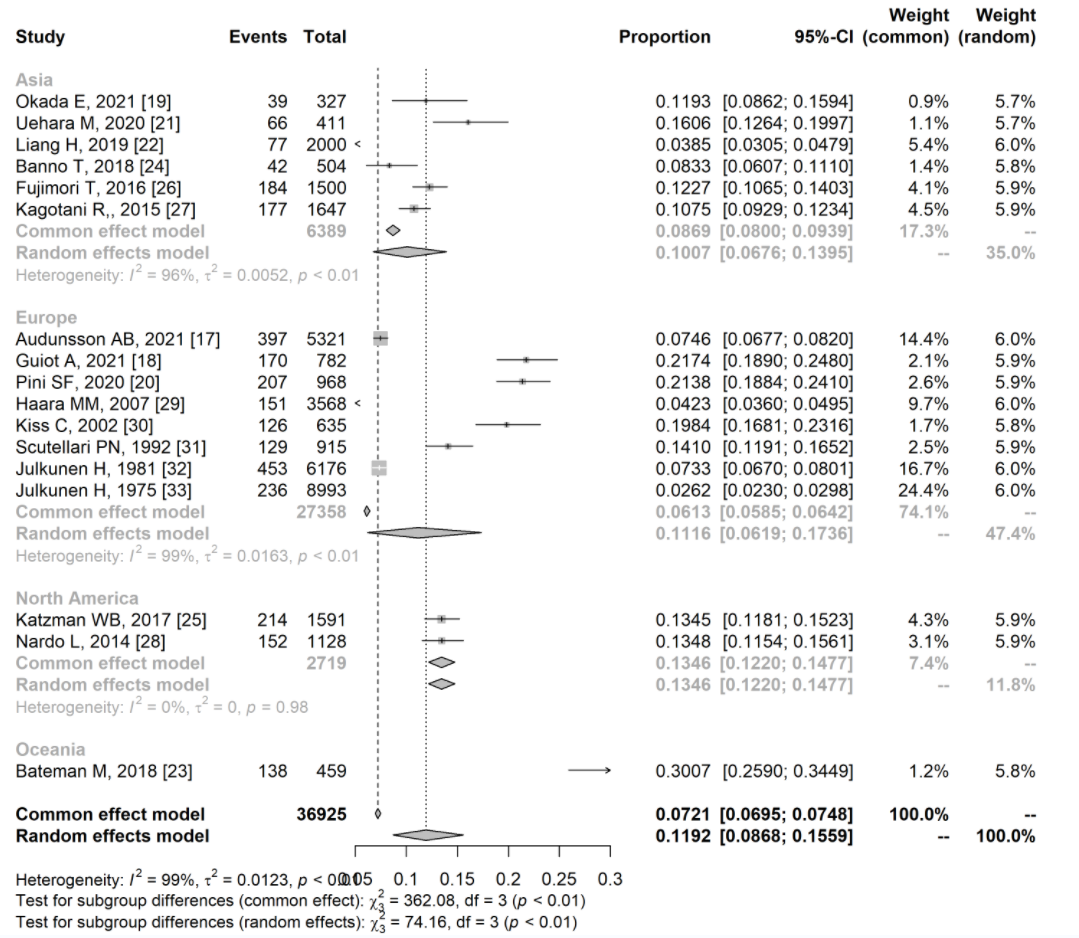


eFigure 4. Forest plot of the prevalence of DISH by continent in clinic-based studies.


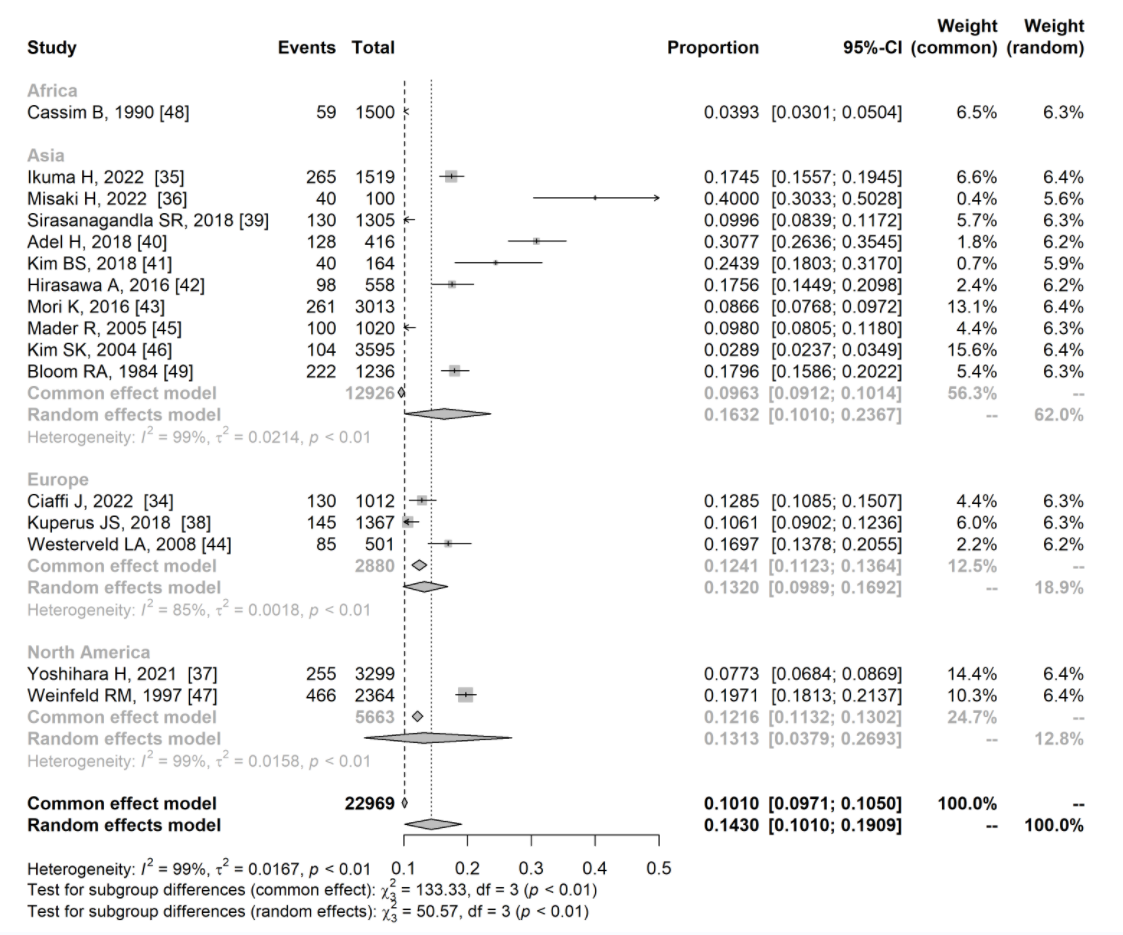


eFigure 5. Forest plot of the prevalence of DISH by sex in population-based studies.


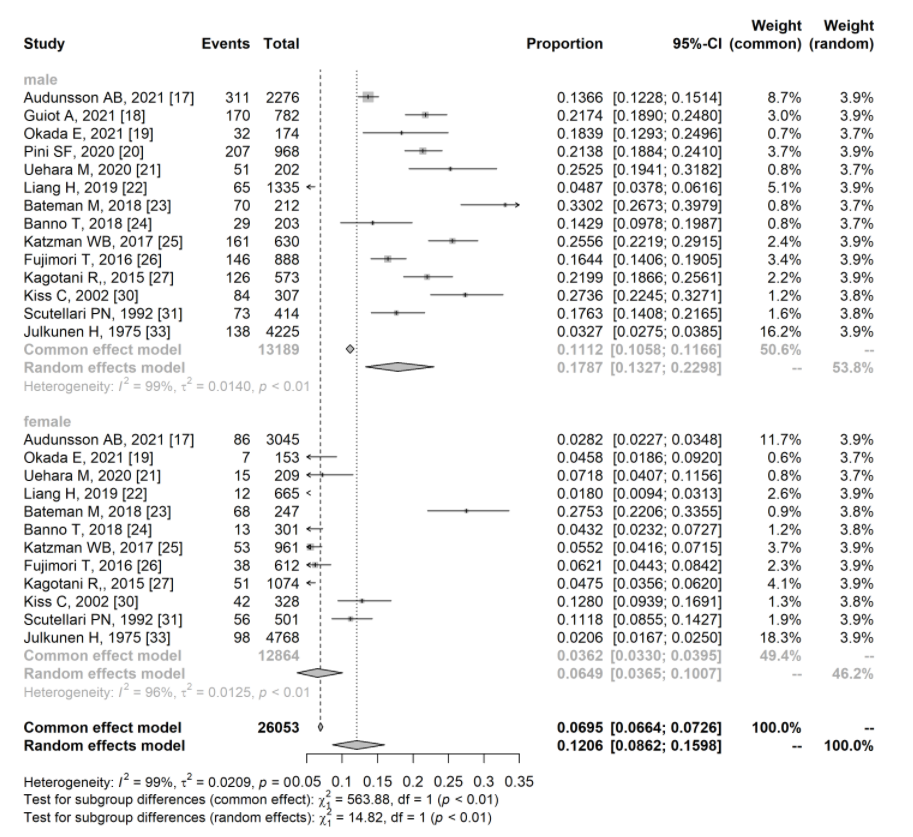


eFigure 6. Forest plot of the prevalence of DISH by sex in clinic-based studies.


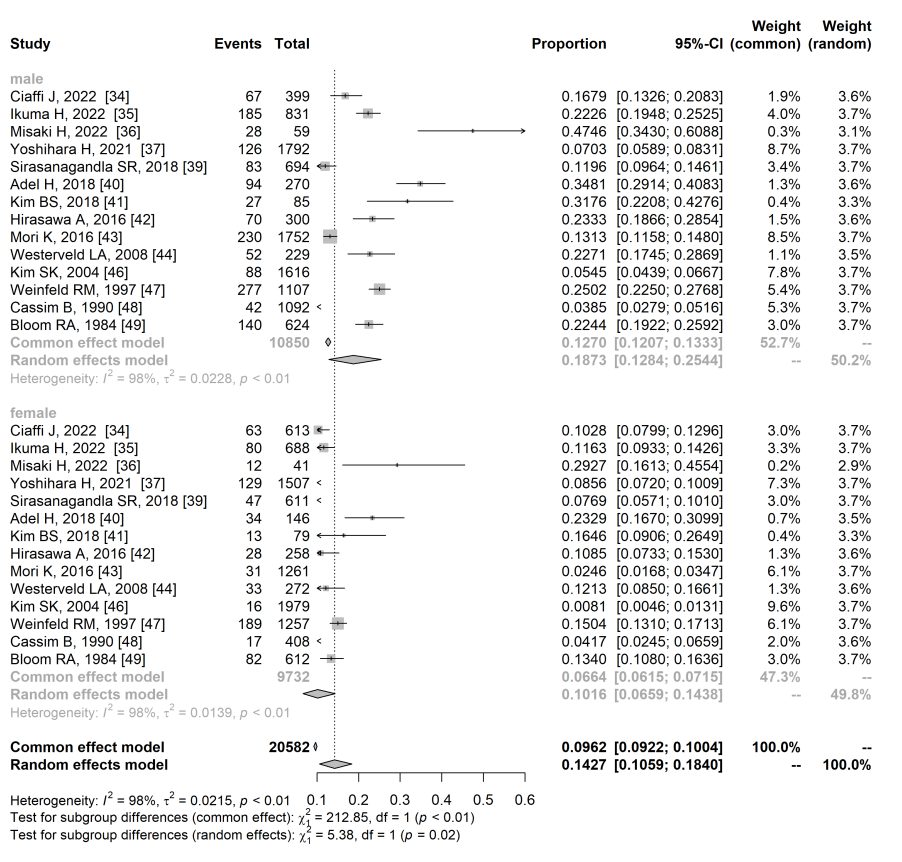


eFigure 7. Forest plot of the prevalence of DISH by race in population-based studies.


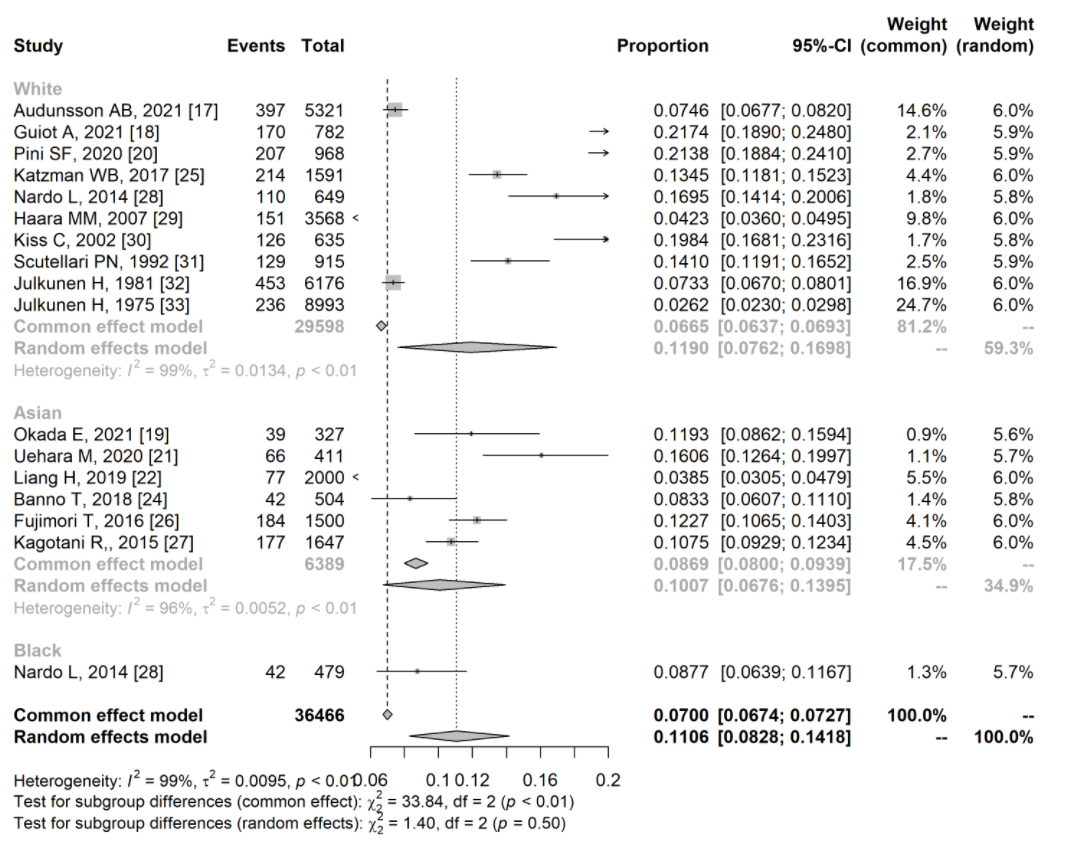


eFigure 8. Forest plot of the prevalence of DISH by race in clinic-based studies.


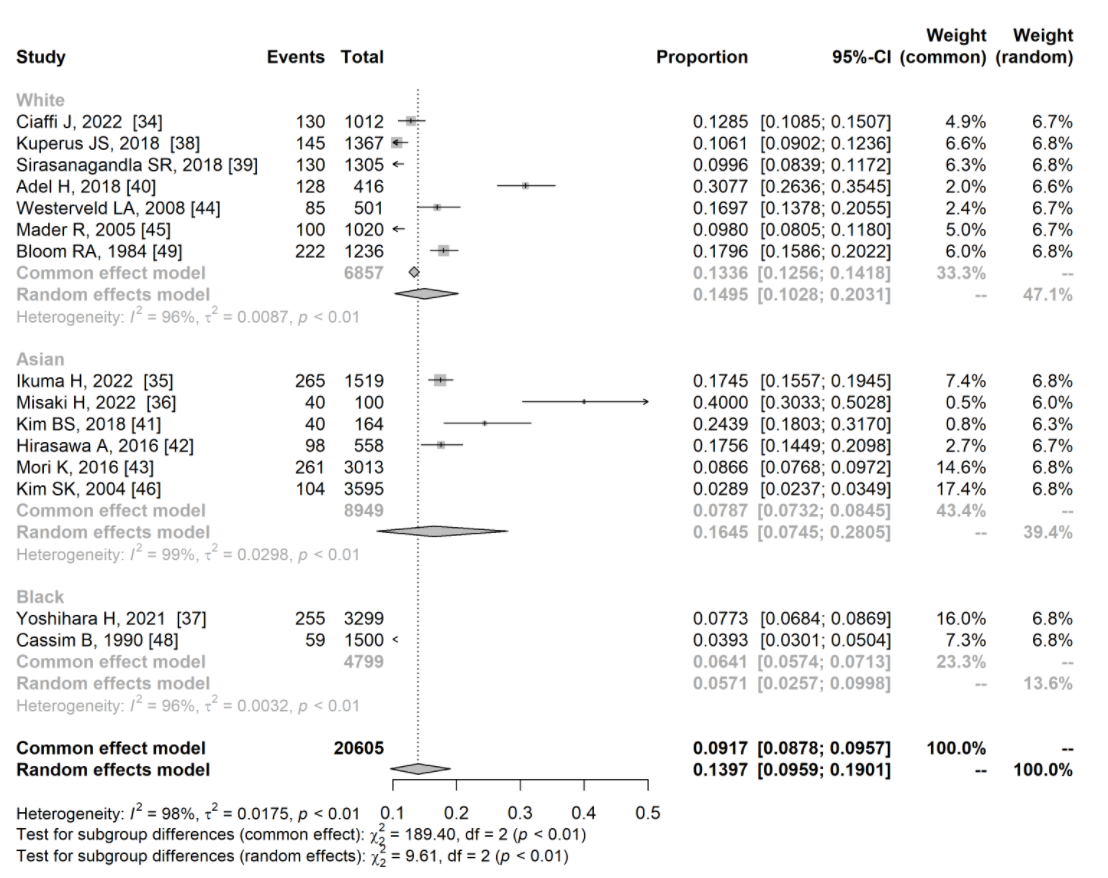


eFigure 9. Forest plot of the prevalence of DISH by age in clinic-based studies.


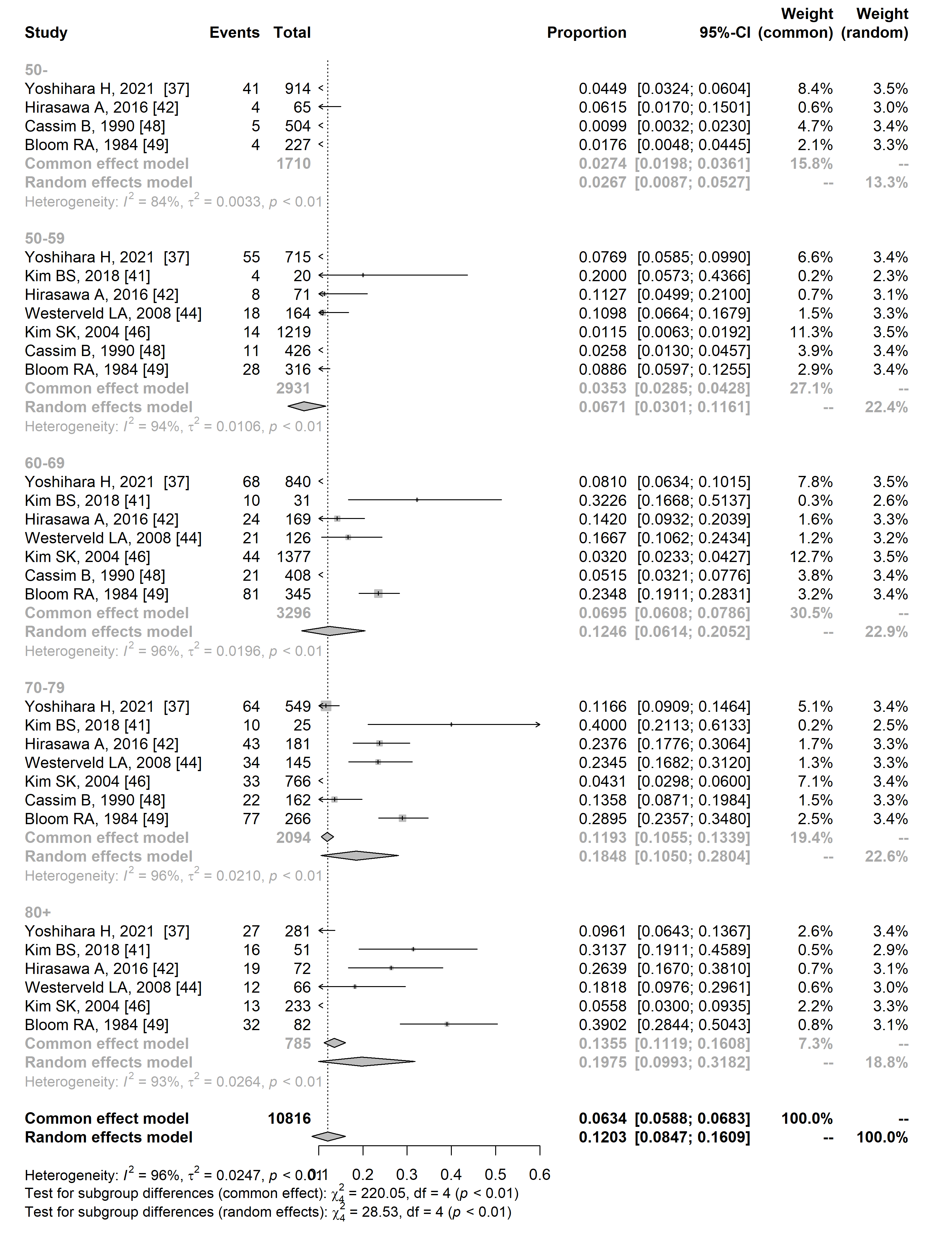


eFigure 10. Publication bias of population-based prevalence studies of DISH.


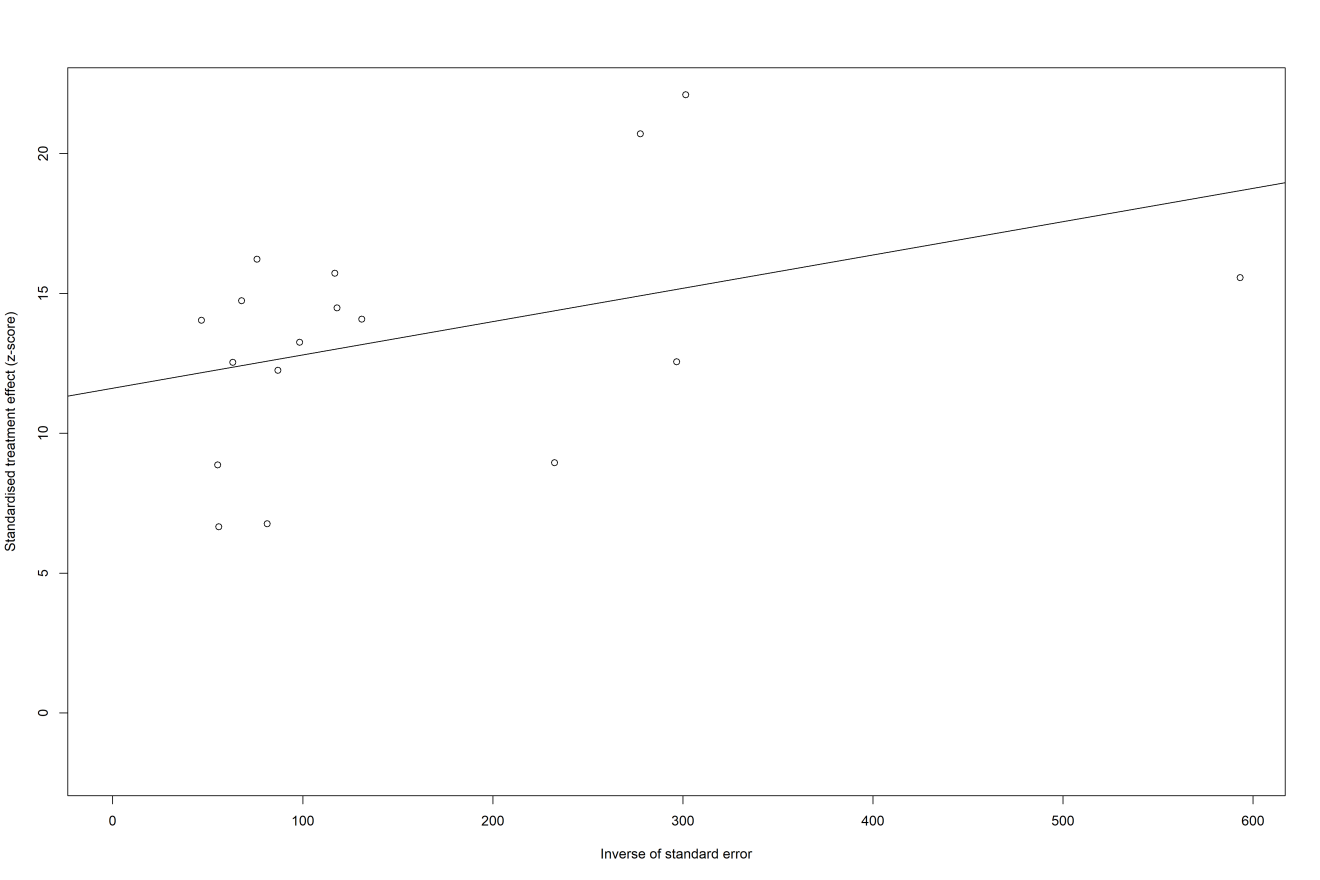


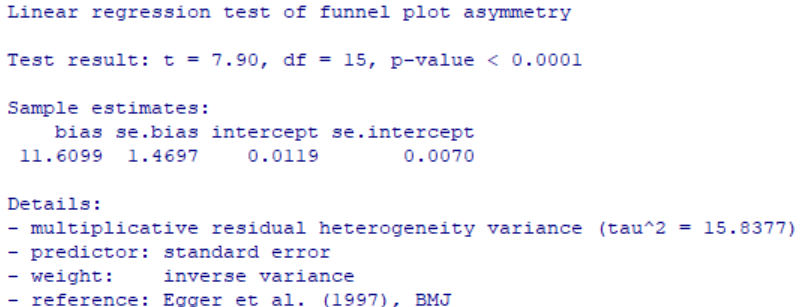


eFigure 11. Publication bias of clinic-based prevalence studies of DISH.


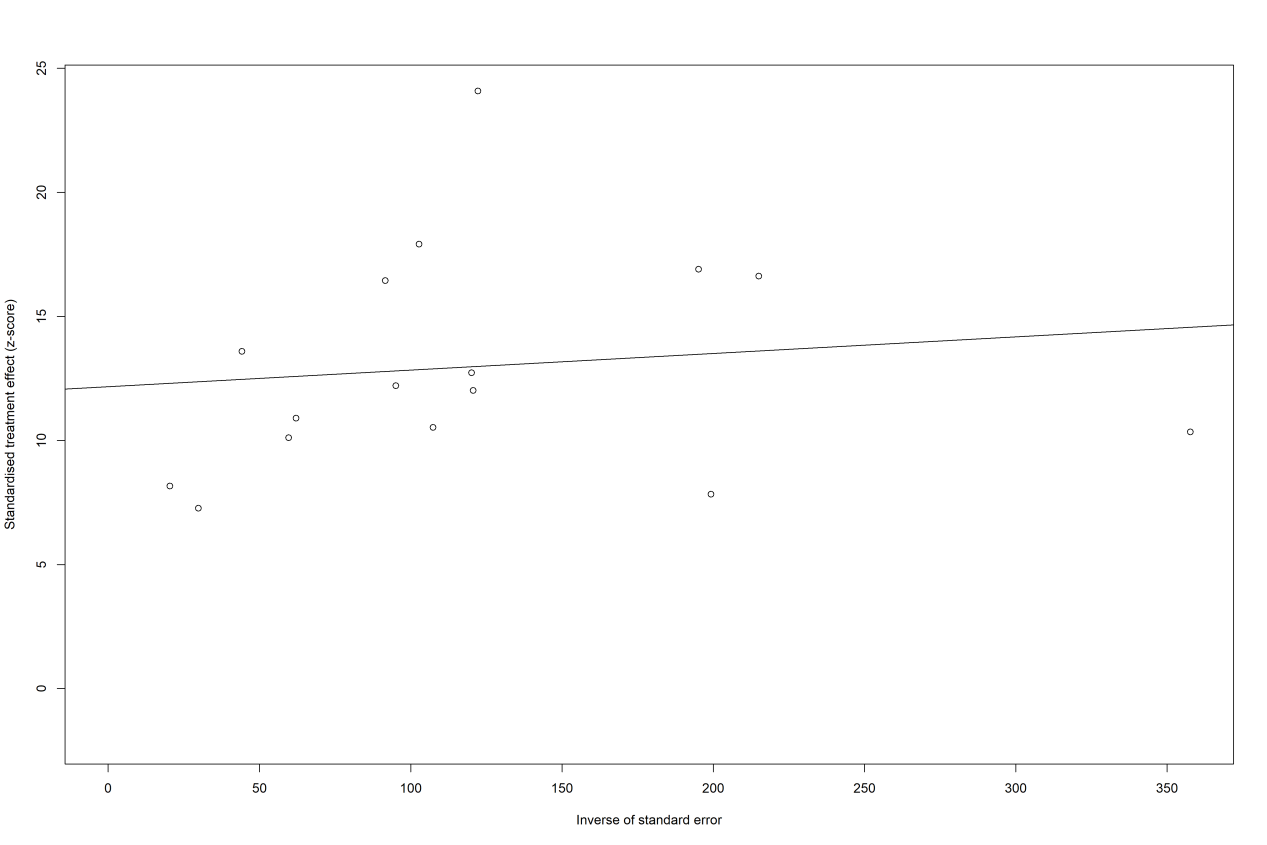


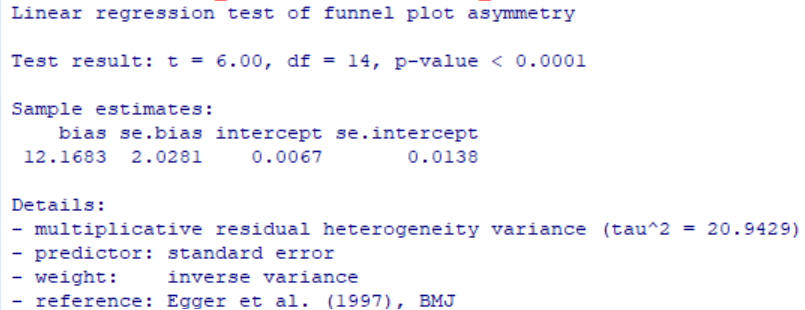

Supplement: Supplementary file 1 [file DataSheet1.docx]
